# Recalibrating Therapeutic Priorities for Duchenne Muscular Dystrophy: A Critical Synthesis of Approved and Emerging Strategies Through the Lens of an Underrepresented Population

**DOI:** 10.3390/genes17070826

**Published:** 2026-07-20

**Authors:** Saken Khaidarov, Aizhan Moldakaryzova, Dias Dautov, Nurgul Sagatbayeva, Banu Yeszhan, Bayan Nurgaliyeva, Saltanat Kenbayeva, Gulban Abdullayeva, Marat Rabandiyarov, Karlygash N. Tazhibayeva, Assel Sadykova, Aibek Yermekbay, Askar Aidarov, Daulet Aidarov, Aray Aidarova, Saniyam Kurbaniyazova, Nazym Abiyrova, Mukhit Kulmaganbetov

**Affiliations:** 1 Department of Molecular Biology and Medical Genetics, Asfendiyarov Kazakh National Medical University, Zheltoksan Street 37A, Almaty 050004, Kazakhstan; khaidarov.s@kaznmu.kz (S.K.); moldakaryzova.a@kaznmu.kz (A.M.); 2 Centre for Orphan Diseases with Neurological Manifestations, Republican Children’s Clinical Hospital “Aksai”, Almaty 050067, Kazakhstan; dz.dautov@gmail.com; 3 Department of Propaedeutics of Childhood Diseases, Children’s City Clinical Hospital No. 2, Asfendiyarov Kazakh National Medical University, Tole-Bi Street 94, Almaty 050012, Kazakhstan; abdullaeva.g@kaznmu.kz (G.A.); rabandiyarov.m@kaznmu.kz (M.R.); 4 Department of Anatomy, Physiology and Sports Medicine, Kazakh Academy of Sport and Tourism, Abay 85, Almaty 050022, Kazakhstan; 5 Department of Propaedeutics of Internal Medicine, Kazakh National Medical University Named After S.D. Asfendiyarov, Tole-Bi Street 94, Almaty 050012, Kazakhstan; nurgalieva.b@kaznmu.kz; 6 Department of Normal Anatomy, Asfendiyarov Kazakh National Medical University, Almaty 050000, Kazakhstan; makhanbetova.s@kaznmu.kz (S.K.); kurbaniyazova.s@kaznmu.kz (S.K.); abiyrova.n@kaznmu.kz (N.A.); 7 Department of Surgery, Faculty of Medicine and Health, Al-Farabi Kazakh National University, Al-Farabi Street 71, Almaty 050040, Kazakhstan; karlygashtazhibay@gmail.com; 8 Almaty Regional Multidisciplinary Clinic, Ministry of Health of the Republic of Kazakhstan, Al-Farabi Street 71, Almaty 050040, Kazakhstan; 9 Department of General Medical Practice, School of Medicine and Healthcare, Al-Farabi University, Al-Farabi Street 71, Almaty 050040, Kazakhstan; aselyasadykova@gmail.com; 10 National Scientific Centre of Phthisiopulmonology, Ministry of Health of The Republic of Kazakhstan 5 Bekkhozhin street, Almaty 050010, Kazakhstan; 11 Department of Anesthesiology and Resuscitation, Asfendiyarov Kazakh National Medical University, Tole-Bi Street 94, Almaty 050012, Kazakhstan; ermekbay.aibek@kaznmu.kz; 12 Department of Oncology, Kazakh-Russian Medical University, Almaty 050012, Kazakhstan; a.askar20@gmail.com (A.A.); daulet_medik@mail.ru (D.A.); 13 Department of Oncology, Asfendiyarov Kazakh National Medical University, Tole-Bi Street 94, Almaty 050012, Kazakhstan; 14 Department of Public Health, Kazakh-Russian Medical University, Abylay Khan Avenue 51/53, Almaty 050040, Kazakhstan; omar.al.askar20@gmail.com; 15 Centre for Eye and Vision Research, Room 901–903, 17W, Hong Kong Science Park, Hong Kong, China; mukhit.k@cevr.hk

**Keywords:** Duchenne muscular dystrophy, therapeutic stratification, precision medicine, exon skipping, gene therapy, ataluren, Elevidys, cost-effectiveness, Central Asia, health policy

## Abstract

Background: Duchenne muscular dystrophy (DMD) has, for the first time, several mutation-specific drugs in clinical use. Four exon-skipping antisense oligonucleotides and one adeno-associated virus (AAV) micro-dystrophin gene therapy carry US FDA approval, and one nonsense-readthrough agent (ataluren) holds a conditional European authorisation that has since been placed under review; ataluren has never been approved by the FDA. Regulatory approval, however, has not translated cleanly into demonstrable clinical benefit, and in resource-limited settings it has not translated into access at all. Methods: We compiled published efficacy estimates for each approved therapy: the EMBARK randomised trial for delandistrogene moxeparvovec (Elevidys), the STRIDE registry for ataluren, and the long-term extension studies of eteplirsen, golodirsen, viltolarsen and casimersen. We read these alongside mutation-spectrum and clinical data from a Kazakh DMD cohort (*n* = 34), one of the first Central Asian populations to be characterised systematically. Because these sources differ in design, population, endpoint and follow-up, the comparison is indirect and descriptive rather than pooled or statistical. On that basis we assembled three descriptive maps: biological versus clinical efficacy, eligibility versus access, and an order-of-magnitude estimate of cost per year of preserved ambulation. Results: Across the approved therapies, larger biological dystrophin restoration did not correspond to longer preservation of walking. We describe this as the absence of the expected positive association rather than as evidence of a negative one: the comparison rests on a small number of drugs whose values come from heterogeneous studies. We did not—and with these data could not—test it as a formal correlation. Ataluren, with roughly 2% dystrophin restoration, was associated with a longer reported delay in loss of ambulation than Elevidys, which restores 34–51% micro-dystrophin. In the Kazakh cohort, 26.5% of patients were biologically eligible for exon-skipping and 11.8% for ataluren, yet realistic access to any mutation-specific therapy was effectively zero as of April 2026. Our order-of-magnitude cost estimates ran from about $12,000 per additional year of ambulation for standard of care to about $1.3 million for AAV gene therapy, a hundredfold range that did not track clinical effect. Conclusions: We argue that the conventional priority ordering (gene therapy first, exon-skipping second, standard care as background) does not hold up when weighed against patient-relevant outcomes and cost, and may reasonably be inverted for resource-limited systems. This is our interpretation of an indirect comparison, not an evidence-based clinical recommendation. On that reading, the highest-value investments for Central Asia are early molecular diagnosis, universal access to glucocorticoids and specialised physiotherapy, and individual-import pathways for ataluren, while AAV gene therapy is, in our view, a lower near-term priority until its durability and safety data improve.

## 1. Introduction

For most of its history, DMD was a disease we understood without being able to treat. The dystrophin gene was cloned in 1987, its pathogenesis worked out through the 1990s, and the reading-frame rule described in 1988 [1], yet the standard of care barely moved for a quarter of a century: glucocorticoids, physiotherapy and respiratory support. A gene this large, in a disease this systemic, seemed beyond the reach of mechanistic intervention.

That changed between 2014 and 2024. Eteplirsen received accelerated FDA approval in 2016—the first mutation-specific DMD therapy and the first antisense oligonucleotide approved for a neuromuscular disorder [2]. Golodirsen, viltolarsen and casimersen followed in 2019, 2020 and 2021, respectively [3,4]. Ataluren obtained a conditional EMA authorisation for nonsense-mutation DMD in 2014 but was never approved by the FDA. In June 2023 the FDA granted accelerated approval to delandistrogene moxeparvovec (Elevidys), the first AAV gene therapy for DMD; approval was expanded for ambulatory patients in 2024, with a simultaneous accelerated approval for non-ambulatory patients that was later withdrawn [5].

A decade of approvals invites an analogy with the targeted therapies that reshaped oncology. We think the available evidence does not support that analogy. The approved drugs are not biologically inert, and most produce a measurable molecular change, but the link between that change and clinical benefit has turned out to be weaker than the regulatory framework assumed. The therapy that restores the most dystrophin is not the one that preserves walking the longest. The therapy with the broadest mutation eligibility is not the one with the most durable clinical signal. And the most expensive therapy is not the one that buys the most additional walking per dollar spent.

These mismatches matter most in the settings where they are least discussed. Central Asia, with a combined population of nearly 80 million, has essentially no access to any approved mutation-specific DMD therapy. Choices made over the next five years, about diagnostic infrastructure, individual-import pathways, compassionate-use programmes, and how much priority to give AAV gene therapy, will decide whether the next generation of patients receives measurable benefit or mainly the prestige of expensive technology of uncertain value.

We set out to do three things. First, to place the biological and clinical efficacy of each approved DMD therapy on one comparative framework and show where molecular and clinical endpoints diverge. Second, to ground that framework in real data from an underrepresented population, using a Kazakh DMD case series (*n* = 34). Third, to propose a therapeutic priority ordering that follows the clinical evidence rather than the chronology of approval. We should be clear at the outset that this is a synthesis and an argument built on indirect comparison, not a head-to-head study, and we return to its limitations in Section 4.6.

## 2. Methods

### 2.1. Data Sources for Therapeutic Efficacy

We drew clinical efficacy estimates for each approved DMD therapy from the strongest available evidence, while recognising that “strongest available” differs markedly by drug. For Elevidys we used the EMBARK phase 3 randomised trial [6] (NCT05096221, *n* = 125) together with post-marketing safety data through November 2025, including the FDA boxed-warning announcement [7] and the EMA refusal of marketing authorisation [8]. For ataluren we used the STRIDE registry interim analysis [9] (data cut-off January 2022, *n* = 307 across 14 countries), propensity-matched to the Cooperative International Neuromuscular Research Group (CINRG) Duchenne natural history study (DNHS). For the exon-skipping oligonucleotides we used the long-term extension studies of eteplirsen, golodirsen, viltolarsen and casimersen [3,4], including the ESSENCE confirmatory trial (NCT02500381). Standard-of-care benchmarks came from CINRG DNHS and the 2018 Birnkrant international care consensus [10].

These sources are not equivalent. They range from one randomised controlled trial (EMBARK) through a propensity-matched registry (STRIDE) to non-randomised, open-label extensions (the exon-skippers), and they differ in population, endpoint definition, and follow-up duration. Any comparison across them is therefore indirect, and we treat the resulting figures as descriptive and hypothesis-generating. The limitations of indirect cross-study comparison are set out in Section 4.6.

Where more than one published estimate existed for a given drug, we chose the estimate from the most recent peer-reviewed analysis of the largest reported population, and where the literature gave a range, we carried that range through rather than collapsing it to a single value. For ataluren, the 2022 STRIDE interim reported 4.0 years of loss-of-ambulation delay and a 2026 propensity-matched update reported 3.5 years; we quote both and use the more conservative 3.5-year figure in the cost estimates. For the exon-skippers we used the 1.5–1.8-year range reported across the extension studies rather than any single most favourable value.

### 2.2. Mutation-Spectrum and Clinical Data from the Kazakh Cohort

The Kazakh DMD cohort was assembled retrospectively from genetic and clinical records at the Republican Children’s Clinical Hospital “Aksai” and Asfendiyarov Kazakh National Medical University (Local Bioethics Committee Protocol No. 01-040325, approved 28 March 2025). Of 40 referred cases, 34 male patients with pathogenic or likely pathogenic DMD variants confirmed by MLPA [11] (SALSA P034-B2/P035-B1 probemix; MRC Holland, Amsterdam, The Netherlands) and/or amplicon-based NGS (NM_004006.2, ≥20× coverage) formed the analytic cohort; the full genetic characterisation of this cohort is reported separately [12]. Six cases were excluded: three with negative MLPA and no NGS, and three in whom NGS identified pathogenic variants outside DMD (SYNE1, CAPN3, and one unresolved). Variants were classified per the 2015 ACMG/AMP guidelines [13] and validated by CENTOGENE GmbH (Rostock, Germany). The cohort is small and drawn from referral centres in a single country, and we treat its eligibility percentages accordingly (Section 4.6).

### 2.3. Therapeutic Eligibility and Access Mapping

For each patient we determined biological eligibility for each approved or experimental strategy using standard mutation-to-therapy criteria: exon-skipping compatibility from the Leiden DMD reading-frame calculator (available online: https://www.dmd.nl/, accessed on 15 April 2026); ataluren eligibility for confirmed nonsense variants without aminoglycoside contraindications; Elevidys eligibility per the November 2025 revised label (ambulatory patients aged ≥4 years without an exon 8/9 deletion); and a CRISPR del 45–55 strategy [14] applied to deletions falling fully or partly within exons 45–55. Real-world access was assessed based on the formal regulatory status of each drug in Kazakhstan as of April 2026 and on the operational availability of individual import pathways.

### 2.4. Cost-per-Outcome Estimation

We estimated cost per additional year of preserved ambulation for each strategy from published US wholesale acquisition prices, reported clinical effect size (years of loss-of-ambulation delay versus matched natural history), and simple duration assumptions (a 10-year treatment horizon for chronic strategies; a single dose for AAV). These are deliberately order-of-magnitude figures for comparative ranking, not health-economic estimates. They rest on US list prices, which overstate net prices in many markets and bear no fixed relationship to prices in Central Asia; they omit administration, monitoring and adverse-event costs; and they use point or range estimates of clinical effect drawn from the heterogeneous sources above. A proper cost-effectiveness analysis for any specific health system would require jurisdiction-specific modelling that we have not attempted. Section 4.6 states these limitations in full.

## 3. Results

### 3.1. The Biological–Clinical Efficacy Mismatch

**Figure 1:**
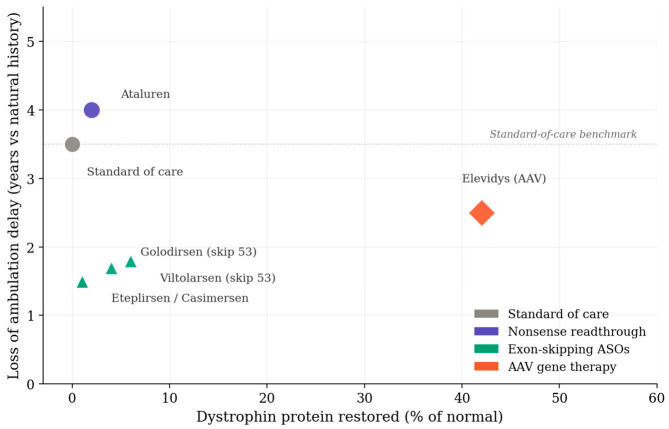
Biological versus clinical efficacy of approved DMD therapies. Horizontal axis: dystrophin protein restored (% of normal) measured in muscle biopsy. Vertical axis: years of loss-of-ambulation delay versus matched natural history (CINRG DNHS or equivalent). The dotted line marks the standard-of-care benchmark of about 3.5 years from glucocorticoids and physiotherapy. The therapy with the highest biological effect (Elevidys, ~42% restoration) shows a smaller clinical effect than a therapy with roughly one-twentieth the biological effect (ataluren, ~2% restoration). The four exon-skipping oligonucleotides cluster near or below the benchmark. Values are drawn from heterogeneous sources (see Methods) and are plotted for descriptive comparison only; no correlation coefficient or trend line is fitted, and the layout is not intended to imply a negative or inverse association.

Plotting biological efficacy (percentage of dystrophin restored) against clinical efficacy (years of loss-of-ambulation delay) for the approved DMD therapies produces a pattern that runs against the usual precision-medicine expectation (Figure 1). We stress that this is a descriptive plot of a small number of drugs whose values come from different study designs. We did not compute a correlation coefficient or test statistical significance, and the figure should be read as a visual summary rather than as evidence of a quantitative relationship.

Three features stand out. First, no approved therapy reaches the upper-right quadrant, where a high biological effect would meet a strong clinical effect. Second, ataluren sits highest on the clinical axis (4.0 years of loss-of-ambulation delay in the 2022 STRIDE interim, *p* < 0.0001; 3.5 years in the 2026 propensity-matched update), above every other approved drug despite the smallest molecular effect among the targeted agents. Third, Elevidys, the most biologically powerful by a wide margin, sits below the standard-of-care benchmark on the clinical axis, and its EMBARK phase 3 trial did not meet its primary endpoint.

The point of the figure is not that any single drug fails, but that the biological surrogate on which most approvals rested (dystrophin restoration) tracks the clinical outcome patients care about (preserved walking) poorly across the class. The exon-skippers were approved on biopsy-measured dystrophin increases of about 1–10% of normal, with confirmatory trials still incomplete or equivocal years later. Elevidys was approved on micro-dystrophin expression of 34–51%, and its post-approval record includes a phase 3 trial that missed its primary endpoint, three deaths from acute liver failure in 2025, an FDA boxed warning and indication restriction in November 2025, and an EMA refusal in July 2025. We do not claim a negative or inverse association between dystrophin restoration and clinical benefit; the data assembled here cannot support a claim of that kind, and we make none. The more modest and defensible reading is that the expected positive association is absent: across these drugs, restoring more dystrophin did not yield greater preservation of walking, and the biological surrogate did not order the therapies as their clinical effects did.

### 3.2. The Eligibility–Access Gap

**Figure 2:**
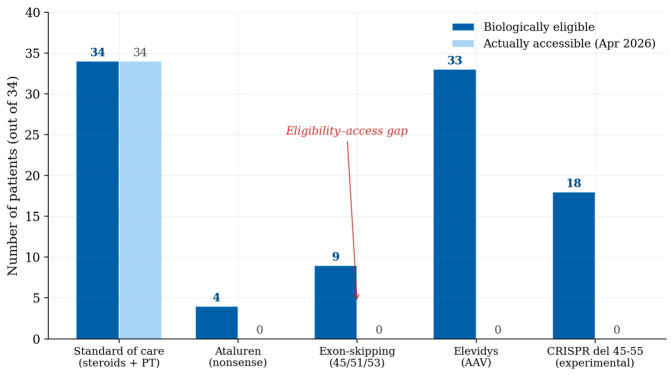
The eligibility–access gap for DMD therapies in the Kazakh cohort (*n* = 34). Dark bars: patients biologically eligible for each strategy by mutation type and other clinical criteria. Light bars: patients who could realistically receive the therapy as of April 2026 given regulatory status, reimbursement and operational access. For every mutation-specific strategy the access bar is zero. Only standard of care shows matching eligibility and access.

Biological eligibility for a mutation-specific therapy is set by the patient’s mutation. Real-world access is set by national drug regulation, reimbursement, supply logistics and the operational existence of import or compassionate-use pathways. The two are often conflated in discussions of precision-medicine readiness. Figure 2 shows what happens when they are separated for the Kazakh cohort.

In biological terms this cohort is unusually well placed for mutation-specific therapy. Deletions cluster in the 44–55 hotspot (78.3% of deletions here, compared with a ~66% TREAT-NMD global average [15]), so 9 of 34 patients (26.5%) are eligible for at least one approved exon-skipping agent, close to double the worldwide figure for eteplirsen alone (~13%). Four patients carry nonsense mutations and would qualify for ataluren. Eighteen have deletions within exons 45–55 that a CRISPR del 45–55 strategy could in principle address. With one exception (a deletion spanning exon 8, the Elevidys contraindication), the remaining 33 would meet the biological criteria for AAV gene therapy. These are small-sample proportions with wide uncertainty, and we treat them as indicative rather than precise (Section 4.6).

The access column tells the opposite story. None of the four exon-skippers is registered in Kazakhstan. Ataluren is technically importable through individual programmes, but the regulatory and logistical infrastructure for those programmes is not yet operational. Elevidys is unaffordable at any plausible national budget allocation, and the November 2025 FDA boxed warning together with the EMA refusal makes its inclusion in a national formulary difficult to defend on benefit–risk grounds even if it were affordable. CRISPR therapy is not in routine clinical use anywhere. A cumulative biological eligibility of about 91% across strategies therefore translates into an access figure that rounds to zero.

This gap is the operational reality that should shape the Kazakh national DMD strategy in 2026 and 2027. The same gap appears across much of Central Asia, Africa, and parts of Latin America and Southeast Asia. It is a structural feature of the current DMD landscape rather than a Kazakh anomaly that faster drug registration alone would fix.

### 3.3. Cost per Year of Preserved Ambulation

**Figure 3:**
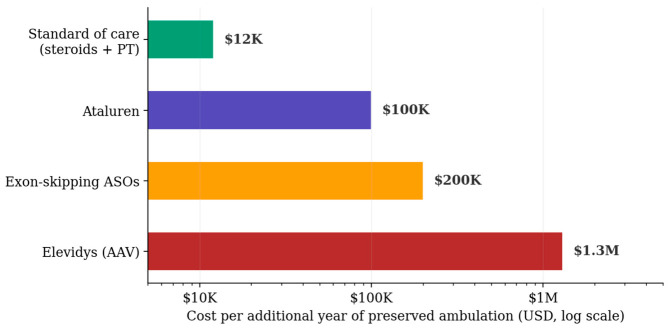
Cost per additional year of preserved ambulation across DMD strategies, on a logarithmic horizontal axis. Standard of care (~$12,000 per year-equivalent) sits about two orders of magnitude below ataluren and exon-skipping ASOs, and more than two orders below Elevidys. The pattern is driven more by the denominator (years of clinical benefit) than by the numerator (drug price). Estimates use US wholesale acquisition prices and published trial efficacy; absolute values vary by jurisdiction, but the relative ranking is more robust than any single value.

Combining clinical effect and acquisition cost into one ratio, dollars per additional year of ambulation, separates the four strategy classes by about two orders of magnitude (Figure 3).

Standard-of-care management of glucocorticoids, physiotherapy and orthoses delivers roughly 3.5 years of ambulation delay at a 10-year cost of about $40,000–$60,000 per patient, or near $12,000 per year of preserved walking. Ataluren delivers about 4 years of additional delay at an annual cost of roughly $300,000–$500,000, or near $100,000 per year of walking. The exon-skippers, at similar annual prices but a smaller effect (1.5–1.8 years in the extension data), come out closer to $200,000 per year of benefit. Elevidys, at $3.2 million for a single dose with a roughly 2.5-year effect, arrives at about $1.3 million per year of preserved ambulation.

This hundredfold spread is central to the policy question rather than incidental to it. For a fixed budget, a health system choosing among these strategies is implicitly choosing between reaching roughly a hundred patients with standard of care, ten with ataluren or exon-skipping, or one with AAV gene therapy. The clinical-effect denominator, not the price tag alone, drives the ranking. As in Section 2.4, these are order-of-magnitude comparisons; absolute values will differ by jurisdiction, and only the broad ranking should be taken from them.

### 3.4. Mutation Distribution and Therapeutic Windows in the Kazakh Cohort

**Figure 4:**
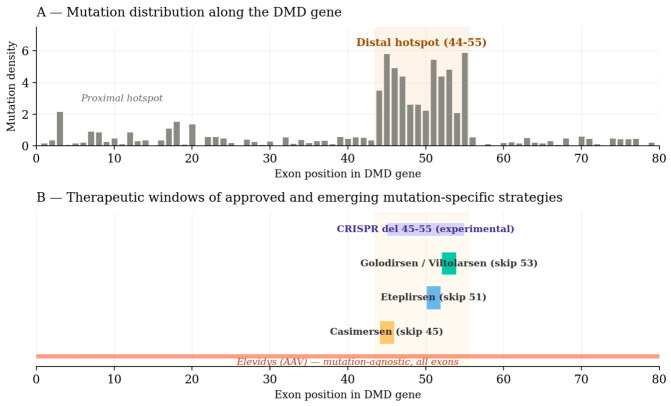
Mutation distribution along the DMD gene (panel (**A**)) and therapeutic windows of approved and emerging strategies (panel (**B**)). The distal hotspot (exons 44–55) holds most deletions in this and most other DMD cohorts. The four approved exon-skippers target exons 45 (casimersen), 51 (eteplirsen) and 53 (golodirsen and viltolarsen), all within the hotspot. CRISPR-mediated deletion of the entire 45–55 region (experimental) would in principle convert any out-of-frame deletion within this envelope to an in-frame Becker-like genotype. Elevidys (AAV) is mutation-agnostic across the gene except for exon 8/9 deletions.

The step from cohort genetics to therapeutic eligibility runs through the alignment between where mutations fall along the DMD gene and where the available strategies act. Figure 4 places these two layers on a common coordinate.

The overlap between the natural mutation hotspot and the therapeutic windows is by design: the exon targets were chosen because deletions cluster there. For populations with a strong distal-hotspot pattern, this produces favourable arithmetic. In our cohort, 78.3% of deletions fall within exons 44–55, a concentration closer to East Asian than to European or Middle Eastern reference cohorts, so cumulative eligibility for any approved exon-skipper is correspondingly higher (26.5% versus the worldwide ~13% for eteplirsen alone). If the access barriers were removed tomorrow, this cohort would sit near the front of the queue for these therapies rather than the back.

A CRISPR del 45–55 strategy, still experimental, would in principle apply to 18 of 34 patients (52.9%) whose deletions fall within the 45–55 envelope, the highest theoretical reach of any strategy short of mutation-agnostic AAV gene therapy. Whether base or prime editing matures into a clinically deliverable form for DMD is a separate question, taken up in the Discussion.

## 4. Discussion

### 4.1. Separating Regulatory Approval, Biological Effect and Clinical Benefit

It helps to keep four things apart that the DMD debate tends to blur: what a regulator approved, what a drug does biologically, what it does clinically, and what it costs. Approval is a regulatory judgement made at a point in time on the evidence then available, and regulators disagree. Both eteplirsen and Elevidys were approved by the FDA (in 2016 and 2023, respectively) on a molecular surrogate, dystrophin expression in muscle biopsy, rather than on a demonstrated clinical benefit, on the reasoning that more dystrophin should mean less degeneration and so preserved walking. The exon-skippers were largely refused by the EMA [2]; Elevidys was refused by the EMA in 2025 [8] while remaining FDA-approved; ataluren took the opposite path, gaining a conditional EMA authorisation it never obtained from the FDA. The regulatory map and the clinical-evidence map are not the same map, and this paper is chiefly about the second.

The accumulating post-approval evidence suggests the surrogate-to-outcome chain is leakier than approval assumed, and the leak sits in a different place for each class. For the exon-skippers it is at the magnitude step: restoring 1–10% of normal dystrophin appears to translate into 1.5–1.8 years of preserved ambulation in the extension data, less than glucocorticoids alone deliver. For Elevidys it is at the structural step: the micro-dystrophin construct restores 34–51% of protein expression, but that protein is truncated to 138 kDa (against 427 kDa for full-length dystrophin) and lacks much of the central rod domain, so “percent dystrophin restored” is a poor proxy for function.

Safety belongs in its own column. Three deaths from acute liver failure in patients given Elevidys or related AAVrh74-platform gene therapies led the FDA to add a boxed warning, restrict the indication to ambulatory patients aged ≥4 years, and require a 200-patient post-marketing study with at least 12 months’ follow-up [7]. The EMA refused authorisation in July 2025 [8], judging that the data did not support a positive benefit–risk balance. Two regulators reading the same evidence reached opposite conclusions, which is itself worth keeping in view whenever approval is used as a proxy for benefit.

### 4.2. Ataluren as an Unexpected Leader

Ataluren’s position at the top of the clinical axis in Figure 1 is, in our reading, the most underappreciated observation in the current landscape, with two large caveats attached. The STRIDE registry (307 patients across 14 countries), propensity-matched to CINRG, reported 4.0 years of loss-of-ambulation delay [9] (*p* < 0.0001), with the 2026 update reporting 3.5 years in a tighter match (median age at loss of ambulation 14.5 years with ataluren versus 11 years with standard of care alone). That is the largest documented delay among the approved drugs.

The caveats are real. STRIDE is observational, and propensity matching cannot remove all confounding; ataluren applies only to the 8–13% of patients with nonsense mutations. But these apply at least as strongly to the alternatives: the exon-skipping extension studies are also non-randomised, and each covers a smaller subgroup than ataluren. Regulatory status adds a further complication that cuts against any simple endorsement. Ataluren was never FDA-approved, its confirmatory ACT DMD trial missed its primary endpoint on the six-minute walk distance, and the EMA has moved to end its conditional authorisation. So the drug with arguably the strongest real-world clinical signal is also the one whose formal approval is weakest and contracting. We read the gap between the negative ACT DMD trial and the more favourable STRIDE data as a lesson about the limits of short randomised trials in a slowly progressive disease, but we do not treat it as settled. It is the reason our Tier 2 recommendation is framed as individual import of an incompletely approved drug rather than as a call for registration.

### 4.3. Why We Would Not Make AAV Gene Therapy a Strategic Priority Now

The case for prioritising AAV gene therapy rests on three claims: that it corrects the underlying defect, that it is a one-time treatment, and that it has the broadest mutation eligibility of the targeted strategies. In our view each needs qualifying.

AAV gene therapy delivers a micro-dystrophin transgene, not the full sequence, and leaves the endogenous mutant gene in place. It supplies an exogenous truncated protein rather than correcting the defect in the sense that gene editing would. Whether that micro-dystrophin persists as muscle fibres turn over, the durability question, remains unresolved years after the first AAV-DMD trials began.

“One-time treatment” understates what the treatment involves. The peri-infusion regimen includes high-dose oral corticosteroids for at least 60 days, weekly liver-function testing for at least three months, weekly cardiac troponin monitoring for at least a month, and access to an immediate-recourse facility for two months afterwards. The single dose sits inside an intensive course closer to oncology induction than to a one-off procedure.

The breadth-of-eligibility claim is technically true but has narrowed. The November 2025 FDA action removed the non-ambulatory indication, and the exon 8/9 contraindication stands. For ambulatory patients with eligible mutations the option remains; for others it has been progressively withdrawn.

Taken together (roughly $3.2 million per dose, substantial monitoring infrastructure, residual safety uncertainty after three deaths, FDA–EMA disagreement, and a phase 3 trial that missed its primary endpoint), this is, in our assessment, not a profile around which a resource-limited health system should build its DMD strategy today. We want to be explicit about the logical status of this statement. Our recommendation that national resources not be committed to AAV gene therapy at present is a policy judgement that follows from weighing cost, safety and durability as we read them; it is not a conclusion that the evidence establishes on its own. The evidence describes a high price, three deaths on the platform, regulatory disagreement and an unmet primary endpoint; the step from those facts to “do not fund this now” is an act of interpretation, and a health system that weighed the same facts differently could reasonably decide otherwise. It is a judgement about near-term priority under budget constraint, not a claim that AAV gene therapy will fail. The approach may well improve; our narrower point is that its evidence base has been contracting rather than strengthening since approval.

### 4.4. A Proposed Priority Ordering (Conceptual Framework)

Two things should be kept apart at this point. The synthesis in the Results is our reading of what the published data describe, and we have tried to keep it close to those data. The ordering proposed below is a policy recommendation that we derive from that synthesis, and it rests on further judgements, about cost, feasibility, monitoring capacity and acceptable risk, that the data alone do not settle. The first is evidence synthesis; the second is interpretation. We present them in that order and label the second as such, so that a reader who accepts our synthesis but weighs the trade-offs differently can part company with us at the right point.

**Figure 5:**
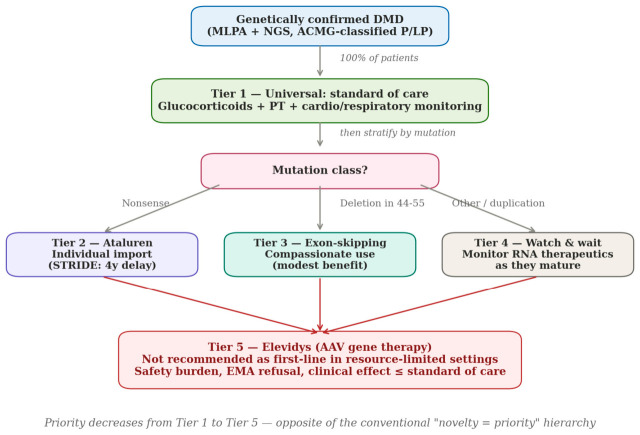
A conceptual priority framework for DMD strategies in resource-limited health systems, proposed by the authors and ordered by strength of clinical evidence and cost-effectiveness rather than by recency of approval. Tier 1 (universal standard of care) gives the largest absolute benefit at the lowest cost and applies to all patients. Tier 2 (ataluren) has the strongest clinical-effect signal among mutation-specific options. Tier 3 (exon-skipping) covers the largest mutation subgroup but with a smaller effect. Tier 4 (watch and wait) is the position for mutation classes not currently served by a high-value approved option. Tier 5 (Elevidys) is placed last. This figure summarises the authors’ argument, not an evidence-based clinical recommendation.

On that basis we propose a priority ordering for DMD strategies in resource-limited systems that follows clinical evidence and cost rather than recency of approval (Figure 5). Figure 5 is a conceptual framework that summarises our argument. It is not a clinical practice guideline, a treatment algorithm, or an evidence-graded recommendation, and it should not be read as one; individual treatment decisions depend on factors outside its scope. Because the ordering rests on the indirect comparison discussed in Section 4.6, it inherits that comparison’s limitations and should be read with the same caution.

Although we frame this ordering for resource-limited systems, the observations behind it are not region-specific. The weak link between dystrophin restoration and preserved walking, and the roughly hundredfold spread in cost per year of ambulation, hold wherever these drugs are used. Higher-income systems face the same value differences but can more easily overlook them, because they can fund several tiers at once rather than choose between them. The reordering we propose is therefore relevant to European and US practice as much as to Central Asia; it is simply harder to see where every option is already available.

Tier 1 is the foundation: glucocorticoids (vamorolone where available, otherwise prednisolone or deflazacort), specialised physiotherapy with KAFO-type orthoses, cardiac protection with ACE inhibitors and beta-blockers as indicated, and respiratory monitoring with non-invasive ventilation once forced vital capacity falls below 50% predicted. CINRG DNHS and the 2018 Birnkrant consensus [10] support this combination as delivering roughly 3.5–4 years of additional ambulation at a 10-year cost near $40,000–$60,000 per patient. Universal Tier 1 access is, in our view, the precondition for any further investment.

Tier 2 is ataluren by individual import for confirmed nonsense mutations—4 of 34 patients (11.8%). The economics compare favourably with the other targeted drugs even at list price, and individual import does not require national registration, though as noted it means importing a drug whose formal approval is weak. We regard it as the highest-value mutation-specific option currently available, with that caveat attached.

Tier 3 is exon-skipping by compassionate-use pathways for eligible deletions—9 of 34 patients (26.5%). The clinical effect is smaller than ataluren’s (about 1.5–1.8 additional years) and the cost per year of benefit higher, but for patients without other options the marginal benefit is real. Compassionate-use arrangements with the manufacturers (Sarepta, Cambridge, MA, USA; NS Pharma, Paramus, NJ, USA) are demanding but precedented.

Tier 4 is a deliberate choice not to act prematurely for mutation classes without a high-value approved option: proximal deletions outside the skipping envelope, duplications, and sequence variants that do not create amenable nonsense codons. For these patients standard of care remains primary while the base-editing, prime-editing and next-generation PPMO pipeline matures.

Tier 5 is Elevidys and placed last. As above, this reflects the current evidence and cost profile rather than a prediction about the technology’s ceiling.

### 4.5. What the Next Generation May Change

The ordering above is calibrated to the evidence available in April 2026. Three pipeline developments could shift it within 5–10 years.

Base editing and prime editing offer the conceptual advantage of CRISPR, permanent correction from a single intervention, without the double-strand breaks that have raised safety concerns about first-generation Cas9 approaches [14]. Roughly 25–35% of DMD patients carry point mutations in principle amenable to base or prime editing. A deliverable platform would widen the addressable population, improve the safety profile relative to AAV gene therapy, and provide the durable correction that micro-dystrophin replacement does not.

Peptide-conjugated PMOs (PPMOs) represent the next-generation chemistry for exon-skipping oligonucleotides, with improved muscle uptake, including cardiac muscle, where current PMOs perform poorly. Phase 1/2 trials of SRP-5051 (Sarepta) began in 2021, and development continues. If a PPMO can restore dystrophin in the 20–30% range rather than the 1–10% range of current PMOs, its clinical effect could approach or exceed that of ataluren.

Multi-exon skipping across the entire 45–55 envelope, rather than one exon at a time, would, in principle, convert a much larger fraction of out-of-frame deletions into an in-frame Becker-like phenotype. The technical requirement, a cocktail of oligonucleotides or a single long antisense molecule blocking several splice-regulatory elements, is non-trivial but actively pursued. Eligibility for such a strategy would be roughly 47% globally and 52.9% in this cohort, against 13% for eteplirsen alone.

### 4.6. Limitations

Several limitations bound what this analysis can claim, and we state them plainly because the argument depends on how the evidence is weighed.

The comparison is indirect, and this is the limitation that most constrains the argument. We set results from a randomised trial (EMBARK), a propensity-matched registry (STRIDE), several non-randomised open-label extensions (the exon-skippers) and post-marketing reports side by side, but these are not interchangeable, and the differences run deeper than the study label. The endpoints are not the same measurement: the six-minute walk distance, the North Star Ambulatory Assessment and time to loss of ambulation capture are related but distinct things, and an effect expressed in one does not convert cleanly into another. The comparators differ, from an internal randomised controlled trial in EMBARK to external natural-history or propensity-matched cohorts elsewhere, and propensity matching leaves residual confounding from unmeasured variables. The populations differ in age, baseline ambulatory function and glucocorticoid regimen. The era differs: standard of care improved across the 2014–2025 span these studies cover, so the natural-history baselines against which each drug was judged are not the same baseline. Registries and open-label extensions are further exposed to selection, survivorship and attrition effects, and to the reporting biases that follow from non-randomised follow-up. Even the biological axis is not measured identically, since dystrophin has been quantified by different assays (Western blot, immunofluorescence) against different reference standards across these drugs.

No statistical procedure we could apply would turn these into a like-for-like comparison. A network meta-analysis, for instance, needs a connected network of shared comparators and commensurable endpoints, and that network does not exist here. We therefore present the comparison as a structured, transparent summary of disparate evidence rather than as a pooled or adjusted estimate, and we read the proposed hierarchy in Section 4.4 with the caution that this status demands: it is a way of organising heterogeneous findings for discussion, not a ranking established by direct evidence.

Figure 1 is descriptive. We plotted biological against clinical effect for a small set of drugs and did not compute a correlation or test significance; with so few points and such heterogeneous sources, a formal correlation would not be meaningful. We therefore describe the pattern as an apparent divergence between the molecular and clinical rankings rather than as a demonstrated inverse relationship, and we have tried to keep to that distinction throughout the paper.

The cost estimates are deliberately coarse. They use US list prices, a fixed treatment horizon, and single or range estimates of clinical effect, and they omit administration, monitoring and adverse-event costs. They are not a health-economic analysis, they are not specific to Kazakhstan or any other market, and only their broad ranking, not their absolute values, should be relied on. A jurisdiction-specific cost-effectiveness model would likely move the numbers, though we would be surprised if it erased a hundredfold spread.

The cohort is small and local. Thirty-four patients from referral centres in one country cannot represent Central Asia, and the mutation proportions we report carry wide confidence intervals; the 26.5% and 11.8% eligibility figures in particular would move appreciably with a few patients either way. The cohort is best read as a concrete instance that the argument has to work for, not as a population estimate.

Finally, the recommendations are ours. The priority ordering in Section 4.4 and Figure 5 is our interpretation of the evidence assembled here, framed for resource-limited settings. It is not an evidence-based clinical guideline, and readers weighing the same sources differently could reasonably order the tiers differently. We have tried to separate what the evidence shows from what we conclude from it, and to flag the second as interpretation wherever it appears.

## 5. Conclusions

The 2014–2024 wave of approvals gave DMD the appearance of a precision-medicine transformation. On the evidence gathered since, the transformation looks more apparent than real. The class that raised dystrophin expression most has not preserved walking the longest; the most expensive option has not bought the most benefit per dollar; the most recently approved drugs are not those with the strongest clinical evidence. We reach these as descriptive observations from an indirect comparison, with the caveats of Section 4.6.

For populations such as those of Central Asia the practical consequence is direct. The Kazakh cohort’s biological eligibility for mutation-specific therapy is high (26.5% for exon-skipping, 11.8% for ataluren, 91% cumulative across strategies) while actual access is close to zero. In our view the useful question is not how to close that gap by importing the newest approved option, but how to allocate limited resources among what demonstrably helps, what might help, and what has a contracting evidence base.

Our recommendations follow, and we offer them as interpretation rather than directive: they are what we would do on the reading above, not what the evidence compels. Build the diagnostic infrastructure that makes mutation-specific therapy possible at all. Provide universal access to the standard of care, which delivers the largest absolute benefit at the lowest cost. Use individual-import pathways for ataluren as the highest-value mutation-specific option, while being clear-eyed about its weak and contracting formal approval. Seek compassionate-use access to exon-skipping for eligible patients. On present evidence we would not commit scarce national resources to AAV gene therapy, though we mean this as a judgement about near-term priority that others may weigh differently, not as a verdict the data impose. And we would watch base editing, prime editing and next-generation PPMOs—the developments most likely to change this calculus.

Figure 5 summarises the argument as a conceptual framework, not a clinical guideline. It reverses the usual ordering in which novelty tracks priority. That reversal argues not against innovation but for letting clinical evidence and cost, rather than the chronology of approval, guide where scarce resources go. For the children in this cohort, and for the far larger number across Central Asia not yet diagnosed, the difference will be measured in years of walking kept or lost.

## Data Availability

De-identified data underlying the analysis are available from the corresponding author on reasonable request, subject to Kazakh national regulations on the protection of patient genetic information.
